# Novel Humoral Prognostic Markers in Small-Cell Lung Carcinoma: A Prospective Study

**DOI:** 10.1371/journal.pone.0143558

**Published:** 2015-11-25

**Authors:** Paul Gozzard, Caroline Chapman, Angela Vincent, Bethan Lang, Paul Maddison

**Affiliations:** 1 Nuffield Department of Clinical Neurosciences, University of Oxford, John Radcliffe Hospital, Oxford, United Kingdom; 2 Division of Medical Sciences & Graduate Entry Medicine, University of Nottingham, Royal Derby Hospital, Derby, United Kingdom; 3 Division of Clinical Neuroscience, University of Nottingham, Queen’s Medical Centre, Nottingham, United Kingdom; University Medical Center of Princeton/Rutgers Robert Wood Johnson Medical School, UNITED STATES

## Abstract

**Purpose:**

Favourable small cell lung carcinoma (SCLC) survival outcomes have been reported in patients with paraneoplastic neurological disorders (PNDs) associated with neuronal antibodies (Neur-Abs), but the presence of a PND might have expedited diagnosis. Our aim was to establish whether neuronal antibodies, independent of clinical neurological features, correlate with SCLC survival.

**Experimental Design:**

262 consecutive SCLC patients were examined: of these, 24 with neurological disease were excluded from this study. The remaining 238 were tested for a broad array of Neur-Abs at the time of cancer diagnosis; survival time was established from follow-up clinical data.

**Results:**

Median survival of the non-PND cohort (n = 238) was 9.5 months. 103 patients (43%) had one or more antigen-defined Neur-Abs. We found significantly longer median survival in 23 patients (10%) with HuD/anti-neuronal nuclear antibody type 1 (ANNA-1, 13.0 months P = 0.037), but not with any of the other antigen-defined antibodies, including the PND-related SOX2 (n = 56, 24%). An additional 28 patients (12%) had uncharacterised anti-neuronal nuclear antibodies (ANNA-U); their median survival time was longer still (15.0 months, *P* = 0.0048), contrasting with the survival time in patients with non-neuronal anti-nuclear antibodies (detected using HEp-2 cells, n = 23 (10%), 9.25 months). In multivariate analyses, both ANNA-1 and ANNA-U independently reduced the mortality hazard by a ratio of 0.532 (*P* = 0.01) and 0.430 (*P*<0.001) respectively.

**Conclusions:**

ANNAs, including the newly described ANNA-U, may be key components of the SCLC immunome and have a potential role in predicting SCLC survival; screening for them could add prognostic value that is similar in magnitude to that of limited staging at diagnosis.

## Introduction

Small-cell lung carcinoma (SCLC) is a high-grade tumour of neuroendocrine origin arising from the lower respiratory tract, primarily affecting older adults, nearly always cigarette smokers; its incidence in the United Kingdom is approximately 8,000 cases per annum [1]. The median survival time with cisplatin/etoposide chemotherapy is 15–20 months for limited stage disease, and 8–13 months for extensive stage disease [2]. The initial response to chemotherapy is typically excellent; however, most patients relapse and are subsequently refractory to treatment. Untreated, or after a relapse, the median survival time is less than 4 months [3].

Evidence of “neuroendocrine” differentiation includes the presence of dense core secretory granules in SCLC cells, with similar ultrastructure to those in Kulchitsky cells; indeed the co-expression of such neurendocrine markers as synaptophysin, chromogranin A and CD56 [4] assists in the immunohistochemical (IHC) diagnosis of SCLC. Moreover, SCLC-associated paraneoplastic neurological disorders (PNDs) are thought to result from auto-immunisation of the host by neural antigens expressed by the neoplastic cells, as exemplified by the Lambert-Eaton myasthenic syndrome (LEMS), a disorder of neuro-muscular transmission we showed to be caused by antibodies against presynaptic P/Q voltage-gated calcium channels (VGCCs) [5]. If the tumour is successfully treated, the LEMS often remits [6].

Another PND, paraneoplastic encephalomyelitis (PEM), is characterised by neuronal loss and inflammatory infiltrates in one or more areas of the nervous system (most frequently dorsal root ganglia, cerebellum and hippocampus) [7]. PEM has a prevalence of 1.6–3.2% in SCLC patients [8]. Unlike LEMS, it is thought not to be antibody-mediated, but over half of all cases have antibodies against the RNA-binding protein ELAVL4 / Hu Antigen D (anti-neuronal nuclear antibody type 1, ANNA-1) [9]. There are two important immunological links between ‘HuD encephalomyelitis’ and cancer. Firstly, whereas ANNA-1/HuD protein expression is normally restricted to neuronal nuclei, it is also expressed by all SCLCs [10]. Secondly, like LEMS, effective treatment of the tumour is an independent predictor of stabilisation of PEM [11].

Favourable tumour outcomes are reported in SCLC patients with LEMS [12] and HuD PEM [13,14]; their neuronal antibodies (Neur-Abs) may be a reflection of more potent anti-tumour immunity. However, these studies are confounded by survival lead-time bias, because the characteristic neurological dysfunction usually prompts earlier cancer screening–often several months before onset of respiratory symptoms [15]. Lead-time bias can be avoided by screening for antibodies in SCLC patients without PNDs, previous such studies have focussed on a narrow range of antibodies, for example in a series of 27 non-PND HuD-Ab positive patients median survival appeared longer for patients with HuD-Abs than their negative counterparts [16]. However, in another similar study, the survival advantage did not correlate with the presence of the HuD or VGCC antibodies themselves [17].

The aim of this prospective study was to determine whether any of a broad range of Neur-Abs (thirteen in total) tested at cancer diagnosis, independent of clinical neurological features, has any impact on survival; the null hypothesis being that none of these antibodies would affect survival.

## Materials and Methods

### Patient Selection and Evaluation

We recruited consecutive patients diagnosed with biopsy-proven SCLC by hospitals in the Nottingham (Trent) region from May 2005 until October 2010 inclusive. At the time of their SCLC diagnosis and before commencement of chemotherapy all patients underwent phlebotomy, and were evaluated neurologically (history and examination), by PM or PG. Further clinical data were obtained from the patient’s medical records, including stage of disease, Karnofsky performance score at tumour diagnosis, any subsequently reported neurological symptoms, and date and cause of death. Patients who reported neurological symptoms after their cancer diagnosis were re-evaluated neurologically by PM. The Euronetwork recommended diagnostic criteria for paraneoplastic neurological syndromes (PNS) [18] were used to define patients with PNS clinically. All patients with PNS were excluded from analysis in this study. We had complete mortality data for all the patients up to 1 May 2012. The Nottingham Research Ethics Committee 2 approved this study, approval reference no. 04/Q2404/100. All participants provided their written informed consent to involvement in the study.

### Serology

Serum was separated by centrifugation and stored at -80°C. Each patient’s serum was tested for the following anti-neuronal antibodies: voltage-gated potassium channel complex (VGKC), P/Q and N-type voltage-gated calcium channel (VGCC), glutamic acid decarboxylase 65KDa isoform (GAD65), leucine-rich glioma inactivated 1 protein (LGI1), contactin-associated protein-2 (CASPR2), NMDA receptor (NMDAR), SOX2, HuD/ANNA-1, amphiphysin (Amph), collapsin response mediator protein 5 (CRMP5), Ri/ANNA-2, Ma2, and Yo. We used the same cut-offs for positivity as in our validated diagnostic clinical assays (+/- 3 standard deviations (SD) of values from 100 healthy control sera). Positivity was confirmed by repeat testing in duplicate. In addition we screened for uncharacterised anti-neuronal nuclear antibodies (ANNA-U) by performing immunohistochemistry (IHC) on primate cerebellum and humanised epithelial cells (HEp-2).

#### Radioimmunoprecipitation assays (RIPA)

VGKC antibodies were detected by immunoprecipitation (IP) of iodinated α-dendrotoxin (^125^-I-α-dendrotoxin)-labelled potassium channel complexes from digitonin-solubilized mammalian whole brain homogenates, as described previously [19]. Titres >100pM were considered positive.

VGCC antibodies were detected by IP of ^125^-I-ω-conotoxin MVIIC labelled P/Q-type VGCCs from digitonin-solubilized mammalian cerebellum, as described previously [20]. Titres >50pM were considered positive.

GAD65 antibodies were detected by IP of ^125^-I-recombinant GAD 65kDa using a commercial kit (RSR Limited, Cardiff, UK). Titres were calculated using the calibrators provided (NIBSC code 97/550); >1,000 arbitrary U/ml was considered positive

#### SOX2 enzyme-linked immunosorbent assay (ELISA)

Autoantibodies to SOX2 were detected by the use of a semi-automated ELISA as described previously [21], using microtitre plates coated with recombinant SOX2 antigen. These were incubated with sera (50μl of 1:100 dilutions in high-salt buffer), followed by washing and addition of horseradish peroxidase (HRP)-conjugated rabbit anti-human IgG/M/κ/λ (Stratech, Soham, UK). Ready-prepared 3,3′,5,5′-tetramethylbenzidine (Chemicon, Chandlers Ford, UK) was used as the chromogenic substrate for HRP, and absorbance values were determined after a 10-minute period at A_650nm_. A cut-off value of an optical density value greater than the mean plus 3SD of the normal population was applied to the autoantibody assay

#### Cell-based assays (CBAs)

LGI1, CASPR2 and NMDAR antibodies were detected by binding to live transfected human embryonic kidney (HEK293) cells expressing the specific antigens, as detailed recently [22,23]. Sera were diluted 1:20 for all except CASPR2, where 1:100 dilutions were used.

#### Immunoblotting

We used commercial immunoblotting to screen for antibodies against recombinant HuD, Yo, Ri, CRMP5, Amph and Ma2 (RAVO Diagnostika, Freiburg, Germany). Samples were diluted 1:2,000 as per the recommended protocol.

#### Immunohistochemistry (IHC)

We tested sera (diluted 1:50) on sections of primate cerebellum (Binding site FS221.A) by indirect immunofluorescence, as per the recommended protocol. All positive sera were screened for non-neuronal anti-nuclear antibodies (ANA) similarly, but using humanised epithelioid cells (HEp-2; Immunoconcepts, Inc. Hep-2000) as per the recommended protocol, with samples diluted 1:100.

### Statistics

Survival data were plotted using the Kaplan-Meier method, and median survival times extrapolated. The uncensored event was death. The Log-rank approach was used to compare survival curves. All univariate analyses were carried out using GraphPad Prism (GraphPad Software Inc, La Jolla, CA) Version 5.00. Multivariate analyses were performed on SPSS (SPSS Inc, Chicago, IL) Version 17.0 for Windows, using the Cox proportional hazards regression model to adjust for known SCLC prognostic factors. Euler diagrams were constructed using the DrawEuler web tool [24].

## Results

### Cohort data

Two hundred and sixty-two SCLC patients were enrolled during the recruitment period (May 2005—Oct. 2010) representing 70% of all newly diagnosed SCLC patients in the Trent region. Twenty-four patients were excluded from this study because they met clinical diagnostic criteria for PND (**S1 Table**), complete details including survival statistics for these patients are published elsewhere [25]. Two hundred and thirty-eight patients were therefore included. Among 238 total subjects, 219 died during follow up period (May 2005 –May 2012). Ninety-four percent of the patients received chemotherapy for their small-cell lung carcinoma. All patients received the same standard universal treatment regimen: cisplatin (or carboplatin) and etoposide. All deaths were from cancer related causes. The median survival time was 9.5 months (range 0.25–77.0 months; **Table 1**). Of the 19 not known to have died, none were lost to follow up and all 19 were censored at the end of our follow up period on 1 May 2012; the median follow up time in the censored group was 53.0 months (range 19.0–77.0 months).

**Table 1:** Study cohort—epidemiological data.

	N	Median survival time in months from Kaplan-Meier analysis (Log Rank Test)
**Total number of patients**	**238** (median age at cancer diagnosis 66yrs, range 43–87), [219 deaths]	9.5, 95% CI* 8.77–10.23,
**Age <65 / age ≥65**	108 (45%) / 130 (55%)	10.25 / 9.0 (*P* = 0.003)
**Female / male**	116 (49%) / 122 (51%)	11.0 / 8.75 (*P* = 0.007)
**Limited stage / extensive at presentation**	86 (36%) / 152 (64%)	14.0 / 8.25 (*P* = <0.001)
**Karnofsky performance score ≥80 at tumour presentation / <80**	98 (41%) / 140 (59%)	11.25 / 8.0 (*P* = <0.001)
**Chemotherapy treated / untreated**	224 (94%) / 14 (6%)	10.0 / 1.25 (*P* = <0.001)
**Cumulative smoking**		
**<15 pack yrs / ≥15 pack yrs**	17 (7%) / 221 (93%)	9.5 / 9.75 (*P* = 0.760)
**<30 pack yrs / ≥30 pack yrs**	62 (26%) / 176 (74%)	9.5 / 9.75 (*P* = 0.989)
**<40 pack yrs / ≥40 pack yrs**	103 (43%) / 135 (57%)	9.25 / 10.0 (*P* = 0.854)

* Brookmayer-Crowley method

**Fig 1:**
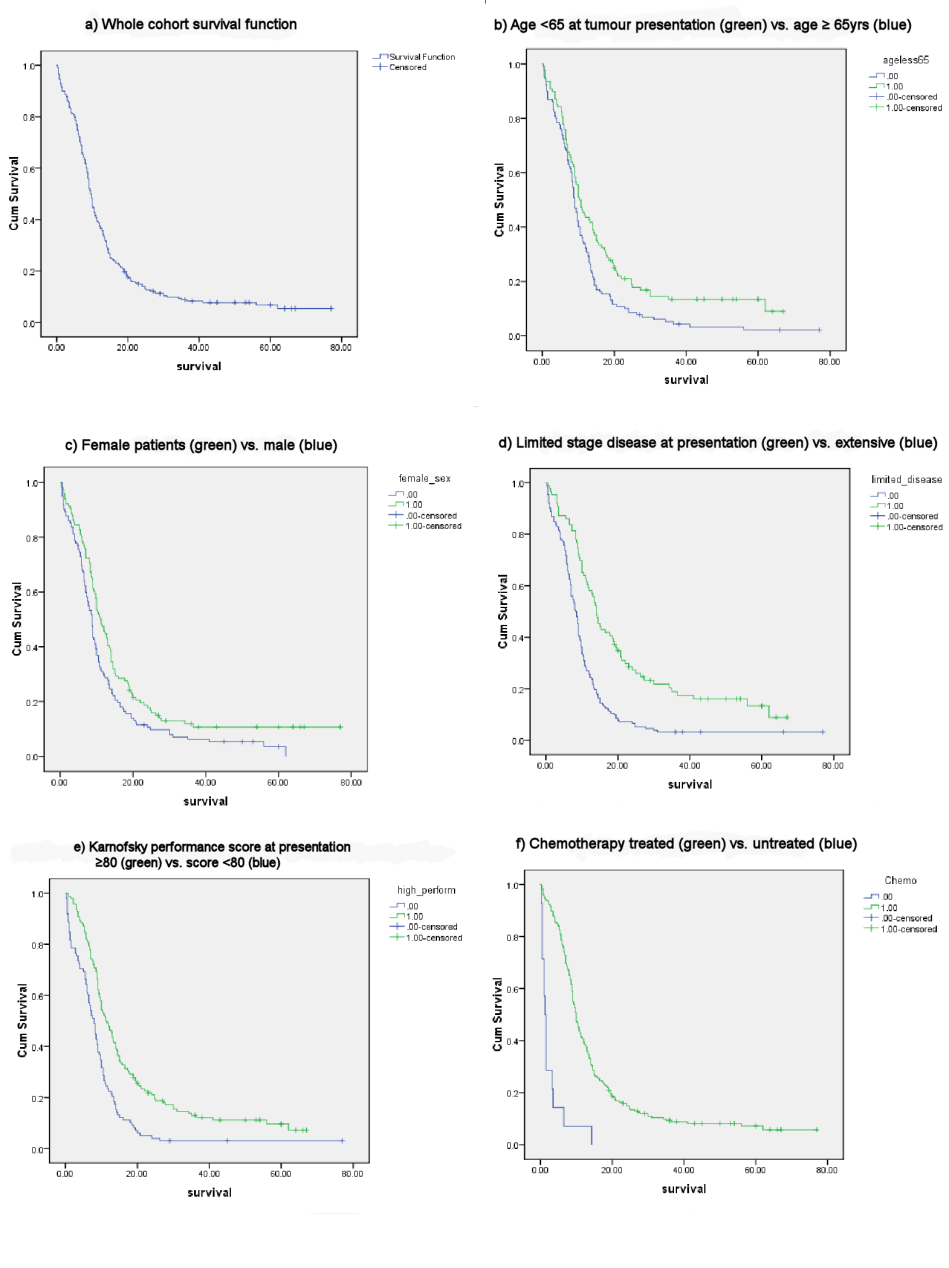
Kaplan-Meier plots: known SCLC prognostic factors. (A) Whole cohort (n = 238) survival function. Subsequent plots compare survival functions for the known prognostic factors: (B) Age; (C) Female sex; (D) Limited disease stage at presentation; (E) Performance score at presentation; (F) Chemotherapy treatment.

As expected, the survival curves dichotomised significantly (Log Rank test) with respect to the known SCLC prognostic factors of age (*P* = 0.003), sex (*P* = 0.003), stage of disease (*P* = <0.001), Karnofsky performance score (*P* = <0.001), and treatment with chemotherapy (*P* = <0.001, **Fig 1**).

### Anti-neuronal antibodies

**Fig 2:**
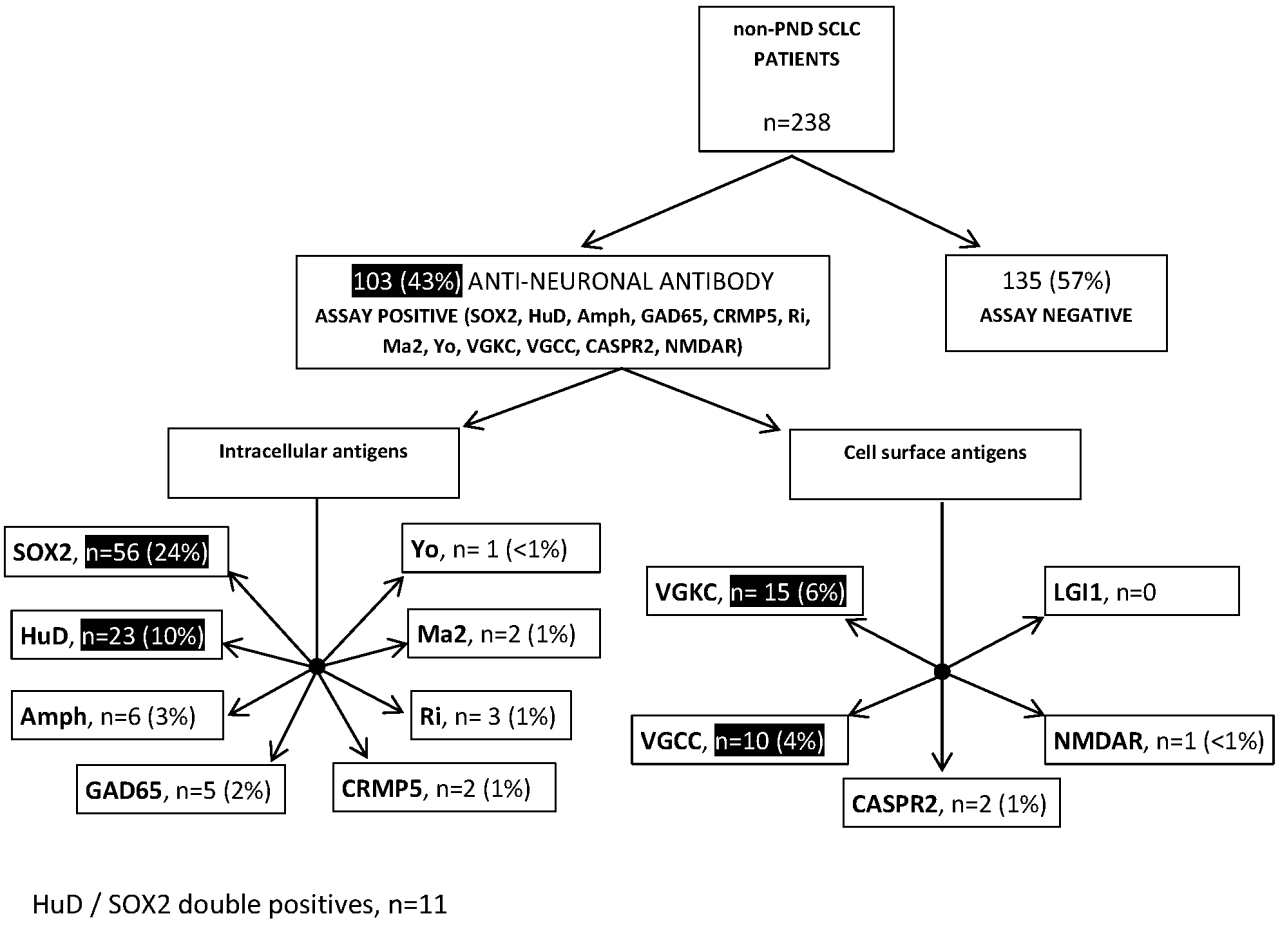
Anti-neuronal antibodies in SCLC patients without neurological disease. 238 SCLC patients who had no evidence of paraneoplastic neurological disease were tested for the following anti-neuronal antibodies: Voltage-gated potassium channel (VGKC), voltage-gated calcium channel (VGCC), glutamic acid decarboxylase 65KDa isoform (GAD65), LGI1, CASPR2, NMDA receptor (NMDAR), SOX2, HuD/ANNA-1, Amphiphysin (Amph), CRMP5, Ri, Ma2, and Yo.

**Fig 3:**
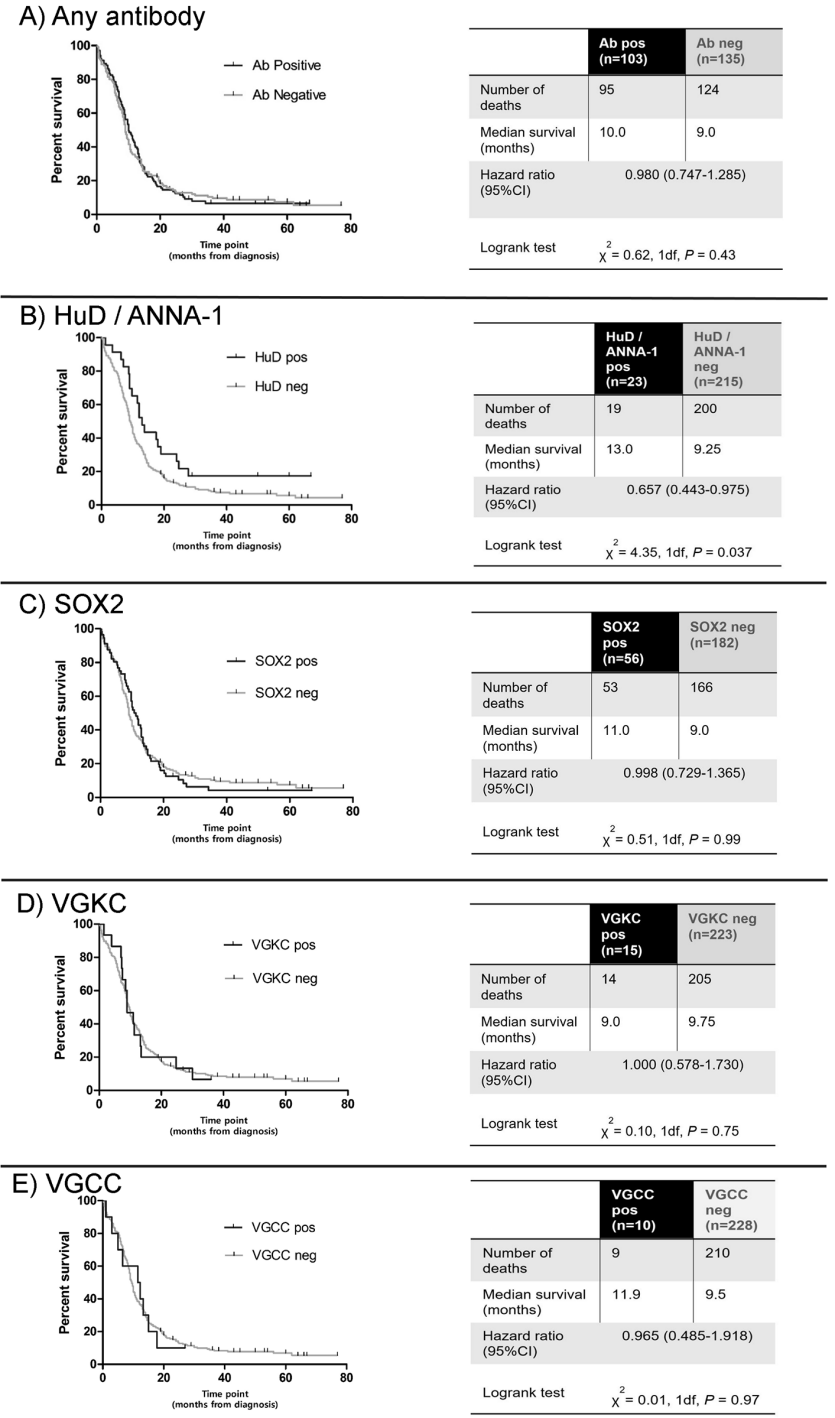
Kaplan-Meier survival curves for antibody positive patients vs. remainder of cohort. (A) Positive in any neuronal antibody assay; (B) HuD / ANNA-1; (C) SOX2; (D) VGKC; (E) VGCC.

One hundred and three patients (43%) tested positive against at least one of the 13 neuronal antigens (**Fig 2**). The most prevalent antibodies were against SOX2 (n = 56, 24%), HuD / ANNA-1 (n = 23, 10%), VGKC (n = 15, 6%), and VGCC (n = 10, 4%). Antibody positive patients, taken together (n = 103), had no significant survival advantage over the rest of the cohort (**Fig 3**). However, when considering each antibody individually, we noted longer survival (13.0 months) in the 23 patients with ANNA-1 / HuD antibodies than in the remainder of the cohort (9.25 months, *P* = 0.037, **Fig 3**), but not for any of the other antibodies, which showed no differences (**Fig 3**). Eleven of the HuD positive patients had coexisting SOX2 antibodies, 2 had coexisting VGKC antibodies, and 1 had coexisting VGCC antibodies. There was no significant survival difference associated with ANNA-1 positive patients who were additionally SOX2 positive (median survival ANNA-1 +ve / SOX2 -ve = 17.75 months, n = 12; ANNA-1 +ve / SOX2 +ve 12.0 months, n = 11, P = 0.4225).

### Anti-neuronal nuclear antibodies (ANNA)

**Fig 4:**
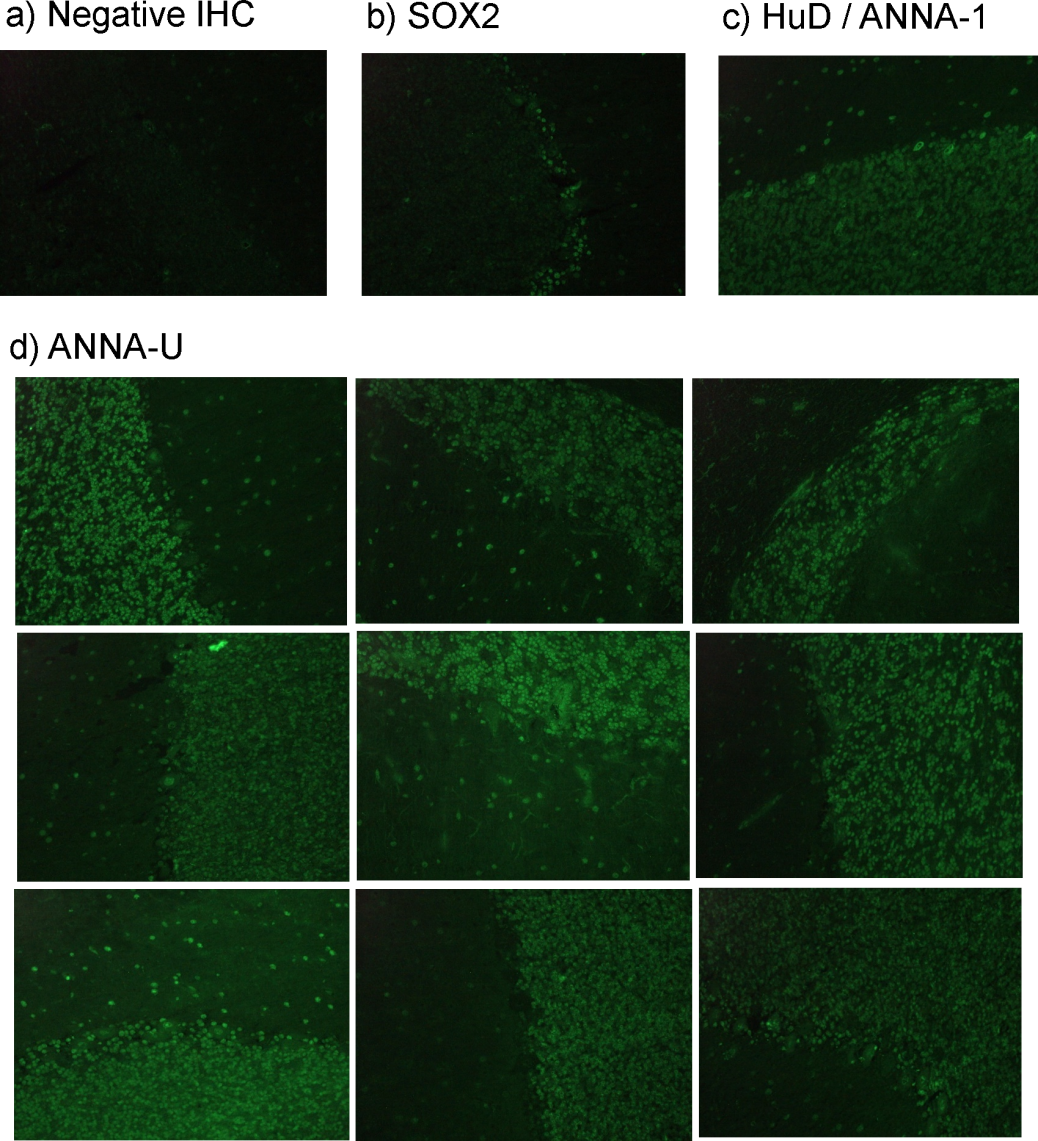
Cerebellar immunohistochemistry (IHC). **X20 magnification.** Positive binding is indicated by green fluorescence. (A) A SCLC patient with negative IHC; (B) SOX2 positive SCLC patient: serum binding is restricted to the Bergmann glia of the molecular laye; (C) HuD / ANNA-1 positive patient: serum binding in a generalised nuclear pattern, staining the densely packed nuclei of the molecular layer plus sparse glial and interneuron nuclei in the molecular layer; (D) Sera from nine representative patients with uncharacterised anti-neuronal nuclear antibodies.

**Fig 5:**
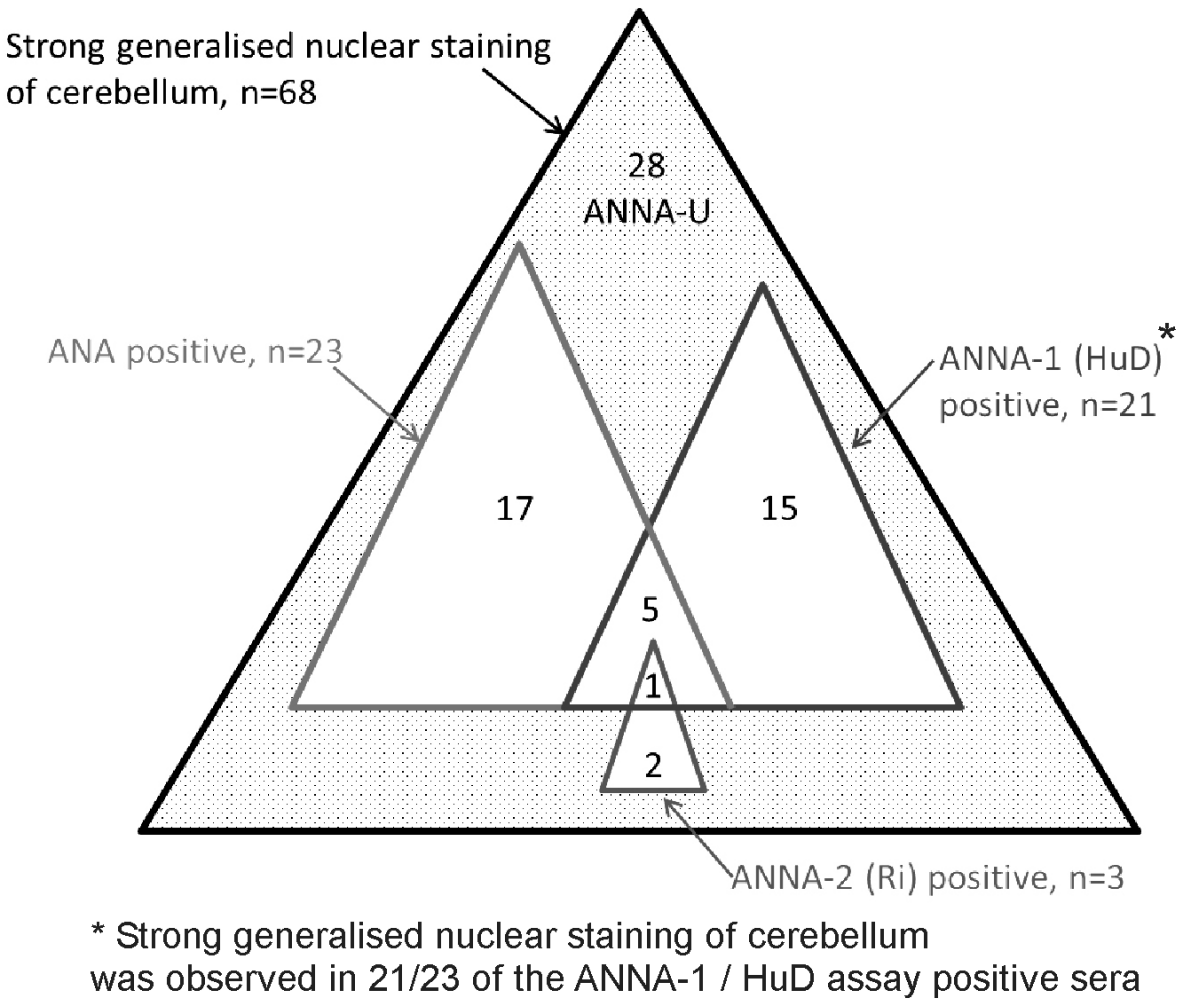
Euler diagram illustrating sub-categories of patients with generalised neuronal nuclear IHC staining. To focus on uncharacterised anti-neuronal nuclear antibodies (ANNA-U), the 68 patients with generalised nuclear staining were tested for the known anti-nuclear antibodies HEp-2 ANA, ANNA-1/HuD, and ANNA-2/Ri; 28 patients were identified who were negative for all three.

To check more broadly for other ANNA besides HuD that might also predict longer survival, we screened all 238 sera for binding to cerebellar sections. We identified 68 sera with strong generalised nuclear staining of cerebellum (at 1:50 dilution), seen as unequivocal and obvious green fluorescence, visible in the entirety of all the nuclei of the granular layer at X20 magnification (examples shown in **Fig 4**). We tested these 68 sera in all the assays for known nuclear antibodies:- 23/68 were HEp-2 ANA positive (i.e. not neuronal specific), 21/68 were positive in the assay for ANNA-1/ HuD, 3/68 were positive for ANNA-2/Ri; 28 were negative in all three assays and thus had uncharacterised anti-neuronal nuclear antibodies (ANNA-U, **Fig 5**).

**Fig 6:**
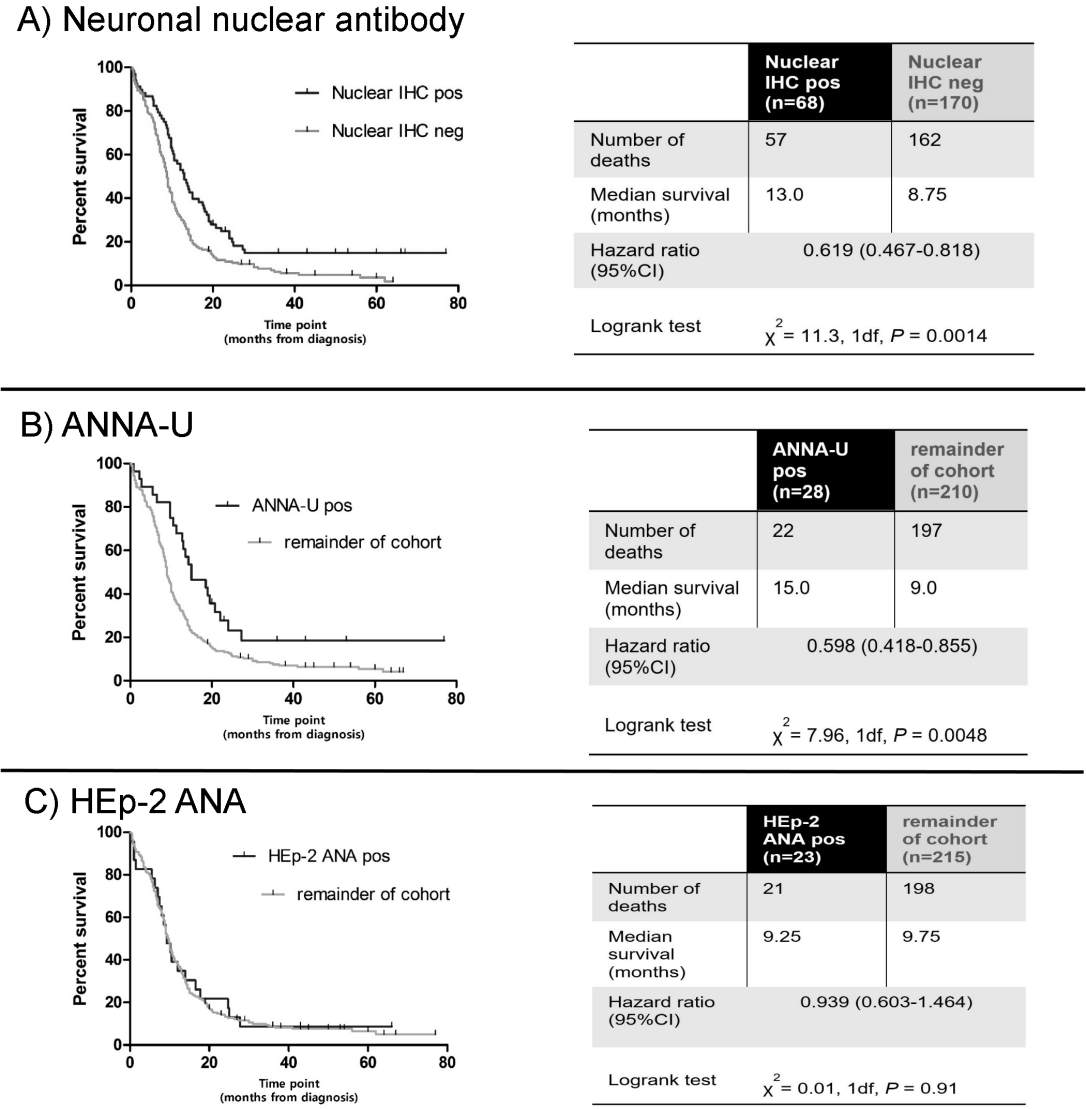
Kaplan-Meier survival curves for patients with neuronal nuclear staining vs. remainder of cohort. (A) The 68 patients with strong generalised nuclear staining on cerebellar immunohistochemistry (IHC) at 1:50 serum dilution; (B) Patients with uncharacterised anti-neuronal nuclear antibodies (ANNA-U); (C) Hep-2 ANA positive patients.

Patients with ANNA-U had a large survival advantage (15.0 months, n = 28, **Fig 6**) over the rest of the cohort (9.0 months; *P* = 0.0048). In contrast there was no survival advantage in the 23 patients with non-neuronal ANAs (detected in HEp-2 cells, 9.75 months, **Fig 6**).

### Multivariate analysis

We performed a Cox regression analysis for ANNA-1/HuD status and ANNA-U, as well as the established SCLC prognostic variables. The reductions in the mortality hazard attributable to both ANNA-1 (Hazard Ratio (HR) 0.532, *P* = 0.01) and ANNA-U (HR 0.430, *P*<0.001) were independent of each other and of the established variables, and highly statistically significant (**Table 2**).

**Table 2:** Multivariate analysis (Cox regression model). ANNA-1 and ANNA-U status were analysed together with established SCLC prognostic factors.

Variables in the equation (univariate analysis *P*-value)	Hazard Ratio [Exp(*b*_*i*_)] (95% CI)	Multivariate analysis *P*-value
**HuD/ANNA-1 (0.037)**	0.532 (0.329–0.860)	.010
**ANNA-U (0.0048)**	0.430 (0.274–0.675)	< .001
**Female sex (0.007)**	0.727 (0.555–0.954)	.021
**Limited disease (<0.001)**	0.446 (0.330–0.602)	< .001
**Chemotherapy treated (<0.001)**	0.130 (0.071–0.238)	< .001
**Karnofsky performance score** * **(<0.001 score <80)**	0.982 (0.974–0.990)	< .001
**Age** * **(0.003 age <65)**	1.015 (1.000–1.031)	.044

Cases analysed 238 (19 censored)

Model fitting information: -2 Log Likelihood 1935.3, Chi-square 138.541, df 7, *P* <0.001

* Performance score and age were analysed as continuous variables in the MVA

## Discussion

In this study of 238 SCLC patients, we observed survival advantages, but surprisingly only in the ten percent of patients known to have antibodies against ANNA-1/HuD (n = 23, 13.0 months, *P* = 0.037), or in the twelve per cent of patients with uncharacterised ANNA (ANNA-U; n = 28, 15.0 months, *P* = 0.0048), and not in those who tested positive for other neuronal targets (even including the strongly LEMS-associated [26] SOX2 (n = 56)), or with non-neuronal ANA (n = 24). In multivariate Cox regression analysis, positivity against ANNA-1/HuD independently reduced the mortality hazard by an HR of 0.532 (*P* = 0.01), and against ANNA-U by HR 0.430 (*P*<0.001), reductions comparable with that for limited disease (HR 0.446; *P*<0.001), and greater than those for female sex (HR 0.727, *P* = 0.021) and Karnofsky performance score (HR 0.982, *P*<0.001).

The sample size was small for the following anti-neuronal antibodies (Amph, GAD65, CRMP5, Ri, Ma2, Yo, CASPR2, NMDAR, LGI1, all n = <6). The impact of these antibodies on survival remains unknown.

The ANNA-1 positive patients that were identified using an antigen specific immunoblot had a significant survival advantage. Since ANNA-1/HuD is an intracellular protein and not expressed on cell surfaces [9], this antibody is unlikely to act directly by killing tumour cells (e.g. through complement activation, antibody-dependent cellular cytotoxicity (ADCC) or opsonisation). ANNA-1 may be a marker of adaptive cell-mediated immunity against SCLC, for example reflecting an underlying CD4+ or CD8+ T-cell response [27,28]. In the elimination phase of cancer immunoediting, specific CD4+ and CD8+ T-cells infiltrate the tumour site and are thought to participate in killing of antigen-positive tumour cells [29]. Indeed, the number of tumour infiltrating lymphocytes in SCLC biopsies predicts clinical outcome significantly [30].

In normal tissue, strong ANNA-1/HuD immunoreactivity is confined to cells of the nervous system and a subset of cells in seminiferous tubules [31]. Since these are both immunologically privileged sites–because of vascular barriers and low HLA expression [32,33]–the HuD in SCLC is effectively tumour-specific, analogous to the well-known cancer-germline antigen NY-ESO-1 [34], which is only normally detected in pre-meiotic spermatogonia and spermatocytes [35] (again with low HLA expression).

We found ANNA-1/HuD positive antibodies in only 10% of SCLC patients (23/238 HuD), despite its expression in all SCLCs [10]. Even though HuD is also normally sequestered from immune cells, many patients may already be tolerant to HuD before their tumours arise, possibly because of tolerogenic presentation of HuD in the thymus–as for many other auto-antigens [36]–or in the CNS (e.g. by microglia) [37] or its draining lymph nodes.

It is noteworthy how many of the patients with strongly positive neuronal nuclear immunohistochemistry were negative in all the known nuclear antibody assays (ANNA-U, n = 28). ANNA-U has no similarity in IHC staining to previously reported undifferentiated anti-neuronal nuclear antibodies (ANNA-3) which binds preferentially to Purkinje cell cerebellar neurons [38]. However, it is likely that ANNA-U staining is due to antibodies targeted at several different uncharacterised antigens. A limitation of this study is that we did not attempt to characterise the ANNA-U antigen(s) at a molecular level. We also note that because ANNA-U was identified on the basis of IHC and negativity for ANNA-1, ANNA-2 and ANA, there could, through overlap, be more patients in the cohort with these antibodies, e.g. some of the ANNA-1 positive patients may also have these uncharacterised antibodies.

We are uncertain as to why a survival advantage was evident in patients with ANNA-U; it is possible that neuronal specific nuclear IHC is an important biomarker of immune mediated destruction of tumour cells. The ANNA-U antibodies in this study can be easily replicated: 1) strong binding to mammalian cerebellar nuclei; 2) no binding to non-neuronal nuclei (HEp-2 ANA); 3) negative in the immunoblot for the known ANNA (ANNA-1/HuD and ANNA-2/Ri). These three screening tests are routinely performed in the evaluation of patients with suspected paraneoplastic disease, and if extended to the wider SCLC population, would yield the valuable prognostic information that is embedded in ANNA-1 and ANNA-U status.

### Translational relevance

In this prospective study of antibody and mortality data in a large cohort of SCLC patients we found evidence of an independent survival advantage associated with anti-neuronal nuclear antibodies (ANNAs), of magnitude comparable to that conferred by the independent prognostic marker of limited stage SCLC disease at diagnosis. We also demonstrated the presence of as-yet uncharacterised ANNAs (ANNA-U), also associated with a significant survival advantage. ANNAs appear to be markers of a protective tumour-specific immune response and may be potential targets for immunotherapy. In clinical practice all SCLC patients could be screened for ANNAs with simple tests that are currently used in the standard work-up for paraneoplastic disorders, namely cerebellar immunohistochemistry and immunoblotting; this would yield valuable prognostic information independent of known markers such as staging and performance status.

## Supporting Information

S1 Table Excluded Patients. The following 24 patients were excluded from the study because they were found to have clinical diagnostic features of a paraneoplastic neurological disorder (PND).
(DOCX)
