# Supplementary material for: Novel Humoral Prognostic Markers in Small-Cell Lung Carcinoma: A Prospective Study
Source: PLoS One. 2015 Nov 25;10(11):e0143558. doi: 10.1371/journal.pone.0143558 (PMC4659625; doi:10.1371/journal.pone.0143558)
Supplement: S1 Table — The following 24 patients were excluded from the study because they were found to have clinical diagnostic features of a paraneoplastic neurological disorder (PND). (DOCX) [file pone.0143558.s001.docx]

| Neurological diagnosis | Age | Sex | Subacute onset (<12 weeks) symptoms & signs, key investigations | PND onset relative to cancer diagnosis  (months) | Cancer staging | Survival (months from cancer diagnosis) |
| --- | --- | --- | --- | --- | --- | --- |
| Lambert-Eaton myasthenic syndrome (LEMS) | 69 | M | *Proximal lower, then upper limb weakness, eyelid droop, dry mouth, dysphagia, slurred speech, proximal limb weakness & reflex potentiation. Neurophysiology fulfilled LEMS diagnostic criteria.* | -6 | Limited | 32 |
| Lambert-Eaton myasthenic syndrome | 63 | F | *Lower limb weakness. Areflexia. Neurophysiology fulfilled LEMS diagnostic criteria.* | -6 | Limited | 36 |
| Lambert-Eaton myasthenic syndrome | 86 | M | *Lower limb weakness, right eyelid droop. Areflexia. Neurophysiology fulfilled LEMS diagnostic criteria.* | -4 | Limited | 11.5 |
| Lambert-Eaton myasthenic syndrome | 74 | M | *Lower limb weakness, dry mouth, constipation. Areflexia and postural hypotension. Patient died before neurophysiology could be performed.* | -2 | Exten. | 1.75 |
| Lambert-Eaton myasthenic syndrome | 60 | M | *Upper and lower limb weakness, dry mouth, limb incoordination, slurred speech. Attenuated reflexes. Cerebellar signs. Neurophysiology fulfilled LEMS diagnostic criteria.* | -3 | Limited | >44 |
| Lambert-Eaton myasthenic syndrome | 53 | F | *Lower limb weakness and incoordination, vertigo. Cerebellar signs. Neurophysiology fulfilled LEMS diagnostic criteria.* | -3 | Limited | 8 |
| Lambert-Eaton myasthenic syndrome | 59 | M | *Upper and lower limb weakness, slurred speech, dry mouth. Neurophysiology fulfilled LEMS diagnostic criteria.* | -1 | Limited | 4.75 |
| Lambert-Eaton myasthenic syndrome | 62 | M | *Lower limb weakness, dry mouth. Areflexic. Sluggish pupillary reflexes. Patient refused neurophysiology.* | -9 | Exten. | 13.5 |
| Lambert-Eaton myasthenic syndrome | 57 | F | *Upper and lower limb weakness, dry mouth, slurred speech. Neurophysiology fulfilled LEMS diagnostic criteria.* | -4 | Limited | 48 |
| Lambert-Eaton myasthenic syndrome | 59 | F | *Proximal upper and lower limb weakness, myalgia, dry mouth. Attenuated reflexes. Postural hypotension. Neurophysiology fulfilled LEMS diagnostic criteria.* | -6 | Limited | >51 |
| Sensory neuronopathy | 58 | M | *Debilitating acral allodynia predominantly affecting the upper limbs.* | +6 | Limited | >30 |
| Sensory neuronopathy | 71 | M | *Peripheral numbness with predominant upper limb involvement. Proprioceptive loss and psuedoathetosis.* | -12 | Exten. | 1.75 |
| Sensory neuronopathy | 53 | F | *Peripheral numbness and gait ataxia. Proprioceptive loss and psuedoathetosis.* | +12 | Exten. | 14 |
| Sensory neuronopathy | 60 | F | *Peripheral numbness and gait ataxia. Proprioceptive loss. Absent sensory nerve conduction studies.* | -7 | Exten. | 18.75 |
| Sensory neuronopathy | 65 | M | *Glove/stocking numbness with sensory ataxia, psuedoathetosis, impaired proprioception and absent reflexes.* | -10 | Limited | 4 |
| Limbic encephalitis | 74 | M | *Memory impairment, temporal lobe seizures. CT head: no metastases* | -1 | Limited | 11 |
| Limbic encephalitis | 75 | M | *Memory impairment, behavioural change, hallucinations, drowsiness. MRI brain: no metastases* | +7 | Exten. | 7 |
| Limbic encephalitis | 66 | F | *Right sided brachiofacial dystonic twitching. MRI brain: no metastases, bitemporal high T2 signal change* | -1 | Exten. | >23 |
| Limbic encephalitis | 70 | F | *Confusion, visual hallucinations, dysphasia. CT head: no metastases* | +2 | Limited | 17.5 |
| Encephalomyelitis (limbic predominant) | 63 | M | *Dementia, personality change, limb numbness and pain, unsteadiness of gait. Postural hypotension. CT head: no metastases* | -13 | Exten. | 9.75 |
| Encephalomyelitis (motor neuropathy predominant) | 69 | M | *Limb weakness and difficulty swallowing. Wasting and fasciculations in all four limbs, bulbar dysarthria.* | -2 | Exten. | 1 |
| Dermatomyositis | 54 | M | *Difficulty walking. Bilateral hip flexion weakness. V sign rash. Gottron’s papules.* | -2 | Limited | 7.75 |
| Dermatomyositis | 50 | M | *Difficulty walking and lifting arms. Weak proximal limb muscles. Heliotrope rash. Creatinine kinase 8000 U/L* | -10 months | Limited | 9.25 |
| Paraneoplastic Cerebellar Degeneration | 54 | F | *Severe unsteadiness of limbs. Gross cerebellar ataxia of all four limbs.* | -2 months | Limited | >57 |

**S1 Table. Excluded Patients:** The following 24 patients were excluded from the study because they were found to have clinical diagnostic features of a paraneoplastic neurological disorder (PND)
